# (+)-JQ1 attenuated LPS-induced microglial inflammation via MAPK/NFκB signaling

**DOI:** 10.1186/s13578-018-0258-7

**Published:** 2018-11-20

**Authors:** Huanhuan Wang, Wenhai Huang, Meihao Liang, Yingying Shi, Chixiao Zhang, Qin Li, Meng Liu, Yikai Shou, Hongping Yin, Xiaozheng Zhu, Xiaoyan Sun, Yu Hu, Zhengrong Shen

**Affiliations:** 1 School of Medicine, Hangzhou Normal University, Hangzhou, China; 2 Institute of Materia Medica, Zhejiang Academy of Medical Sciences, No. 182, Tianmushan Road, Hangzhou, 310013 China; 3 School of Information Science and Engineering, Hangzhou Normal University, Hangzhou, China

**Keywords:** (+)-JQ1, Microglia, Lipopolysaccharide, MAPK, NFκB

## Abstract

**Background:**

Microglia activation is a crucial event in neurodegenerative disease. The depression of microglial inflammatory response is considered a promising therapeutic strategy. NFκB signaling, including IKK/IκB phosphotylation, p65 nucelus relocalization and NFκB-related genes transcription are prevalent accepted to play important role in microglial activation. (+)-JQ1, a BRD4 inhibitor firstly discovered as an anti-tumor agent, was later confirmed to be an anti-inflammatory compound. However, its anti-inflammatory effect in microglia and central neural system remains unclear.

**Results:**

In the current work, microglial BV2 cells were applied and treatment with lipopolysaccharide (LPS) to induce inflammation and later administered with (+)-JQ1. In parallel, LPS and (+)-JQ1 was intracerebroventricular injected in IL-1β-luc transgenic mice, followed by fluorescence evaluation and brain tissue collection. Results showed that (+)-JQ1 treatment could significantly reduce LPS induced transcription of inflammatory cytokines both in vitro and in vivo. (+)-JQ1 could inhibit LPS induced MAPK but not PI3K signaling phosphorylation, NFκB relocalization and transcription activity. In animal experiments, (+)-JQ1 postponed LPS induced microglial and astrocytes activation, which was also dependent on MAPK/NFκB signaling.

**Conclusions:**

Thus, our data demonstrated that (+)-JQ1 could inhibit LPS induced microglia associated neuroinflammation, via the attenuation of MAPK/NFκB signaling.

## Background

Neuroinflammation is an inherent host-defense mechanism to protect and restore the normal structure and function of the brain against infection and injury [1]. In neurodegenerative diseases, neuroinflammation initially clears up infection to control the disease severity and progression. However, neuroinflammation also acts as a double-edged sword [2]. On one hand, neuroinflammation induces and/or aggravates neurodegeneration in the central nervous system (CNS), while on the other hand, it favors the recovery of the injured neurons [3]. Excessive and chronic inflammatory responses in CNS are believed to lead to deleterious effects involving immune cells, brain cells and signaling molecules. Neuroinflammation is considered to induce and accelerate pathogenesis of many neurodegenerative diseases, like Parkinson’s disease (PD), Alzheimer’s disease (AD) and multiple sclerosis (MS). Thus, neuroinflammatory pathways are indicated as promising therapeutic targets for these diseases [4].

Microglia is resident cells of the brain that regulate brain development, maintenance of neuronal networks, and injury repair. Microglia serve as the major cellular component of the innate immune system in the brain and are tightly regulated by the CNS microenvironment [5–7]. They are responsible for the elimination of microbes, dead cells, redundant synapses, protein aggregates, and other particulate and soluble antigens that may endanger the CNS. In response to a wide variety of insults, these cells shift to an activated phenotype that is necessary for the proper restoration of brain homeostasis. However, when the intensity of a threat is relatively high, microglial activation worsens the progression of damage rather than providing protection, with potentially significant consequences for neuronal survival [8]. Furthermore, as the primary source of proinflammatory cytokines, microglia is pivotal mediators of neuroinflammation and can induce or modulate a broad spectrum of cellular responses [9].

Activated microglia is involved in damage-associated molecular patterns of neuronal damage and environmental toxins, such as β amyloid (Aβ) peptide, lipopolysaccharide (LPS) and lipoteichoic acid (LTA). Several signaling pathways play important roles in regulating microglia related neuroinflammation. NFκB (nuclear factor kappa-light-chain-enhancer of activated B cells) signaling activation in microglia results in an increased expression of a series of inflammation-related genes [10, 11]. NFκB-dependent inflammatory gene expression is regulated at multiple levels, including cytoplasmic signaling events leading to the nuclear translocation of NFκB, binding of nuclear NFκB to various transcription factors or regulators, and the posttranslational modifications of histones and NFκB [12]. Within the nucleus, NFκB recognizes the cognate NFκB sites on the enhancer or promoter regions of its target genes and directs the binding of coregulators to form the transcriptional machinery for target gene expression [13]. Targeting NFκB-mediated activation of microglia could provide a novel strategy for treating neuroinflammation.

Bromodomain is an approximately 110 amino acid protein domain that interacts with chromatin, such as transcription factors, histone acetylases and nucleosome remodeling complexes [14]. The bromodomain-containing protein 2 (BRD2), BRD3 and BRD4 proteins share the similar structural features and hence are known as BET (bromodomain and extrater-minal) family proteins [15]. BET domain coregulators are believed to play important roles in obesity, inflammation and cancer [14]. BRD proteins promote inducible gene transcription by the recruitment of transcriptional co-activators including positive transcription elongation factor b (TEFb) [16]. Increasing studies have reported the significant role of BRD4 in the pathology of inflammatory diseases. BRD4 not only regulates the expression of many NFκB-dependent inflammatory genes [17–19] but also participates in the expression of inflammatory gene enhancer RNA (eRNA) synthesis [20, 21] in an acetylated lysine-dependent manner [22]. These data indicate that BET bromodomain has the potential to affect inflammatory responses.

(+)-JQ1 is a small-molecule BRD4 inhibitor devised based on the structure of the Mitsubishi compound that binds to bromodomains, preventing them from reading acetyl-lysine residues and modulating transcription [23]. Earlier studies revealed that (+)-JQ1 and other BET inhibitors disproportionately suppress specific genes and exert potent effects on cancer proliferation [23–29]. Then several studies have shown that (+)-JQ1 might also participate in tissue fibrosis [30, 31]. Later (+)-JQ1 has been described as anti-inflammatory agents in various disease-related local inflammations, including rheumatoid arthritis (RA), MS, periodontitis, psoriasis, colitis, airways, kidney and cardiovascular inflammation [20, 32–38].

Evidence has been shown that (+)-JQ1 treatment resulted in a significant down-regulation of key inflammatory genes in LPS-activated macrophage cells via TLR4 [39]. Then Jung et al. [40] reported that (+)-JQ1 treatment resulted in the significant downregulation of key inflammatory genes in LPS-activated BV-2 microglial cells, implying the contribution of BET bromodomain to neuroinflammation. However, in vivo phenotypes from (+)-JQ1 treated animals remain inconsistent. In one study, BRD4 was proved to be necessary for memory formation in neurons [41], while others reported that BRD4 inhibition was able to enhance cognitive performance in AD mice, which may also be relevant with glial-activation and neuroinflammation [42, 43]. Nevertheless, the specific signaling pathways and underlying mechanism participating in (+)-JQ1 treatment induced inflammatory inhibition remains to be defined. Therefore, in the current study, BRD4 inhibitor (+)-JQ1 will be applied and its influence in modulating glial activation related neuroinflammation and the underlying mechanisms will be carefully examined.

## Results

### (+)-JQ1 has no effect on BV-2 cell viability

**Fig. 1:**
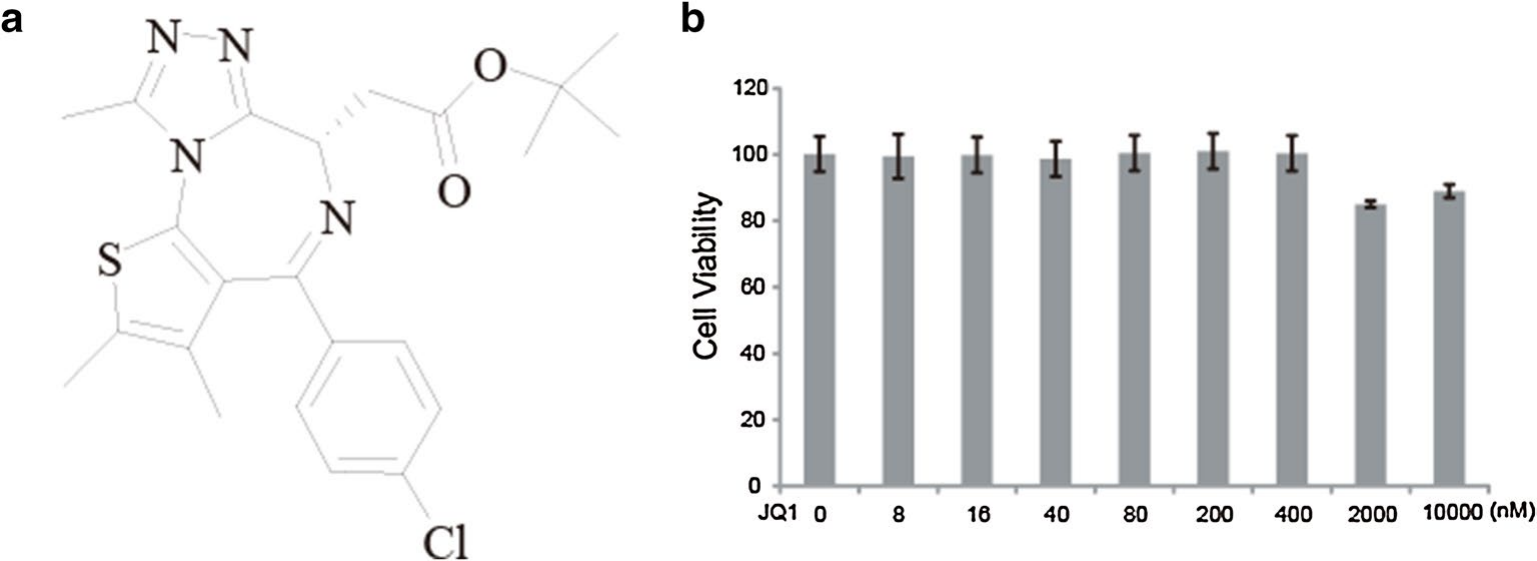
Effect of (+)-JQ1 on cell viability of microglia BV2 cells. **a** The chemical structure of (+)-JQ1. **b** BV2 cells were treated with 0–10000 nM (+)-JQ1 for 24 h, and cell viability was determined by the MTT assay. The data are presented as the mean ± SEM (n = 3)

(+)-JQ1 is a kind of BRD4 inhibitor, whose structure containing benzodiazepines fragment (Fig. 1a). Some research reported that (+)-JQ1 displays anti-inflammatory properties. Thus, we sought to determine the effect of (+)-JQ1 on microglial activation and the subsequent inflammatory response in CNS. First, the effect of (+)-JQ1 on the viability of BV2 microglia was evaluated using the MTT assay, which revealed that BV2 microglia treated with (+)-JQ1 at concentrations of up to 10 μM displayed no significant change in cell survival compared to the control cells (Fig. 1b). Therefore, this concentration range was used for the subsequent functional studies of (+)-JQ1.

### (+)-JQ1 reduces expressions of LPS/Aβ-induced proinflammatory cytokines in microglia

**Fig. 2:**
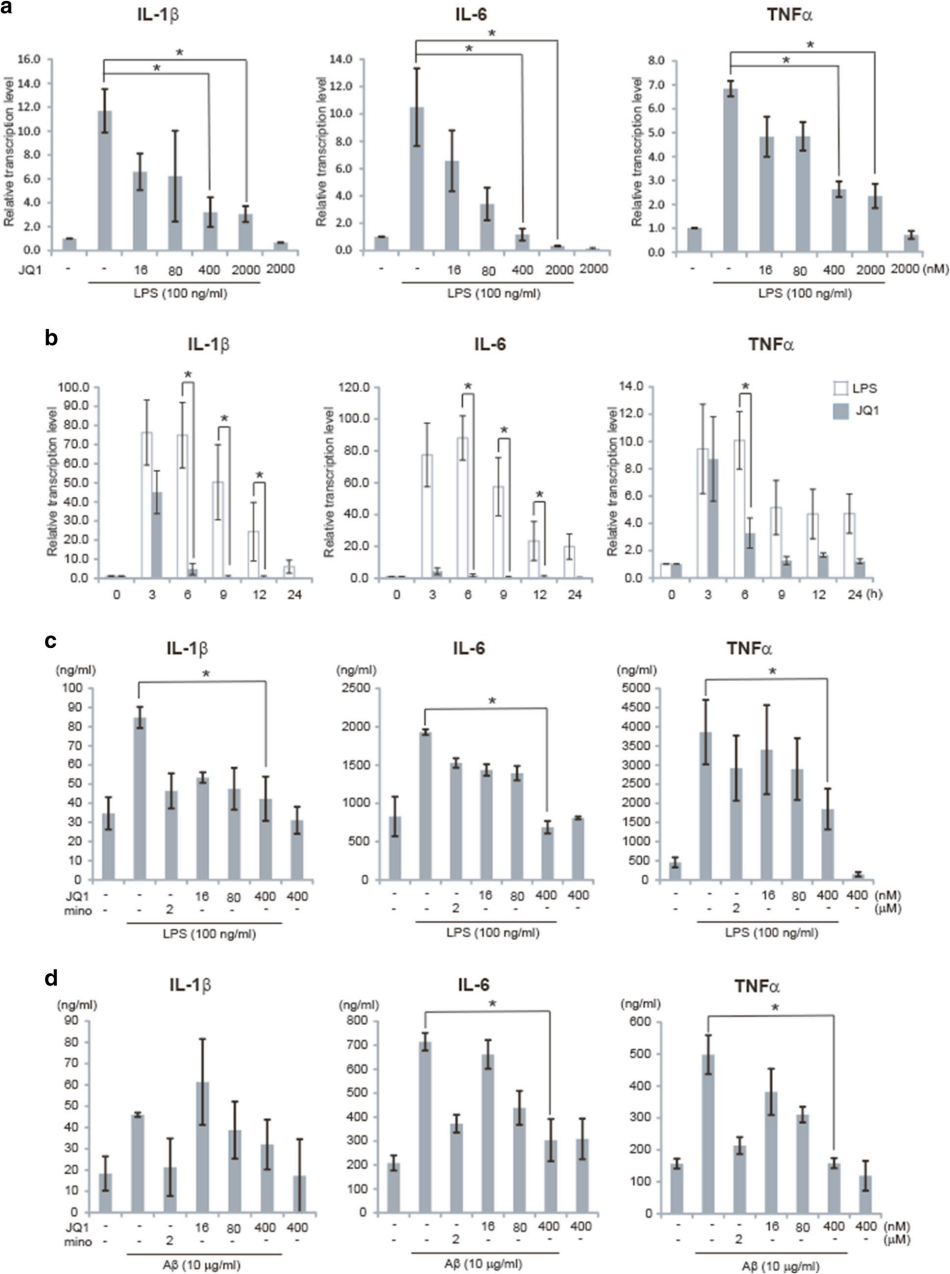
(+)-JQ1 reduces expressions of LPS/Aβ-induced proinflammatory cytokines in microglia. **a** BV2 cells were pretreated with (+)-JQ1 or the vehicle (DMSO) at the indicated dose, followed by LPS (100 ng/ml) stimulation for 4 h. **b** Alternatively, BV2 cells were pretreated with either (+)-JQ1 (400 nM) or DMSO, followed by LPS stimulation for the indicated period. After incubation, the cells were collected and lysed for quantitative analysis of transcription levels of IL-1β, IL-6 and TNFα by qRT-PCR. **c**, **d** BV2 cells were pretreated with (+)-JQ1, DMSO, or minomycine (mino) at the indicated dose, followed by LPS (100 ng/ml) or Aβ (10 μM) stimulation for 24 h. IL-1β, IL-6 and TNFα levels in the culture medium were determined via ELISA. The data are presented as the mean ± SEM (n = 3, * *p *≤ 0.05)

Activated microglia is known to produce a large quantity of proinflammatory cytokines and other factors that cause neuronal damage and cytotoxicity in CNS [44]. Thus, we evaluated the role of (+)-JQ1 in the microglial response to LPS stimulation. BV2 cells were preincubated in (+)-JQ1 (0–2000 nM) for 30 min and then subjected to LPS stimulation. The results revealed that JQ1 suppressed the LPS-induced transcription of IL-1β, IL-6 and TNFα by microglia in a dose-dependent manner (Fig. 2a). The regulation of cytokine transcription by (+)-JQ1 remained effective throughout the period of the measured LPS incubation, particularly at the early phase (Fig. 2b). Concomitantly, the secretion of IL-1β, IL-6 and TNFα induced by LPS released from BV2 cells were down-regulated upon exposure to (+)-JQ1 in a dose-dependent manner (Fig. 2c). Minomycine (2 μM) was administrated as the positive control. Besides, Aβ was proven to induce microglial activation and serve as an etiological factor of neurological disorders [44]. Then, we investigated whether (+)-JQ1 could suppress Aβ-induced proinflammatory cytokine expression and found that pretreatment with (+)-JQ1 produced a significant dose-dependent decrease in IL-1β, IL-6 and TNFα production in response to Aβ (Fig. 2d). These results suggest the negative regulation of (+)-JQ1 on the LPS/Aβ-stimulated microglial proinflammatory response.

### (+)-JQ1 attenuates NFκB phosphorylation, translocation and transcription in microglia

**Fig. 3:**
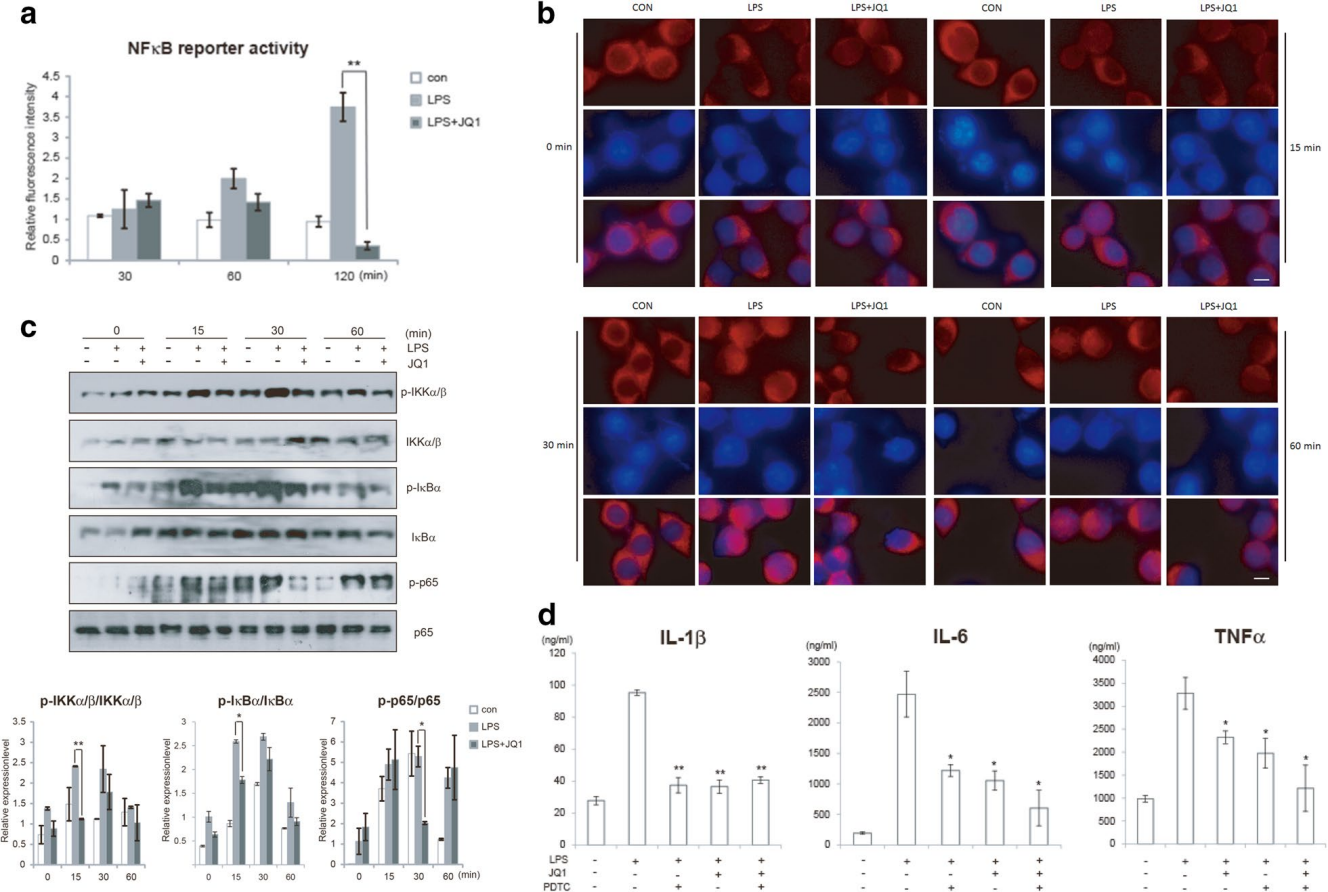
(+)-JQ1 attenuates NFκB phosphorylation, translocation and transcription induced by LPS. **a** BV2 cells were transfected with either pGL3-NFκB or pGL3 along with pRL-TK. After 24 h, the cells were treated with either DMSO or (+)-JQ1 (400 nM) followed by LPS (100 ng/ml) stimulation. NFκB-mediated promoter activity was assessed by a luciferase assay (n = 3). **b** p65 nuclear translocation upon LPS (100 ng/ml) stimulation in either the presence or absence of (+)-JQ1 (400 nM) was detected by immunofluorescence staining and confocal microscopy. p65 immunoreactivity is shown in red, and nuclei were stained with DAPI (blue). (Scale bar = 20 μm). **c** BV2 cells preincubated with either (+)-JQ1 (400 nM) or DMSO were stimulated with LPS (100 ng/ml) for the indicated periods. Phosphorylation of IKKα/β (p-IKKα/β), IκBα (p-IκBα) and p65 (p-p65), as well as the total protein content, were analyzed by immnoblot. Representative results from three independent experiments are depicted here. The relative intensity of a given band was normalized (n = 6). **d** BV2 cells were treated with LPS (100 ng/ml) or JQ1 (400 nM), together with or without PDTC (p65 inhibitor). 24 h later, cell culture medium were collected and IL-1β, IL-6 and TNFα were tested by ELISA (n = 3). The data are presented as the mean ± SEM (* *p *≤ 0.05, ** *p *≤ 0.01)

NFκB signaling and its corresponding kinase phospohorylation are play important role in the regulation of pro-inflammatory cytokine transcription. Due to the pivotal role of (+)-JQ1 in the regulation of the production of proinflammatory cytokines in LPS-stimulated microglia, we then examined whether the biochemical activity of (+)-JQ1 occurred via the modulation of NFκB signaling. Treatment with (+)-JQ1 generated a reduction in NFκB activity (NFκB reporter gene experiment) in LPS-stimulated BV2 cells (Fig. 3a). It is known that the loss of repression of p65 from the IκBα restraint and the subsequent translocation of p65 to the nucleus are key steps in the initiation of gene transcription [45]. Thus, we examined the effect of (+)-JQ1 on p65 translocation in LPS-activated microglia. As shown in Fig. 3b, robust nuclear translocation of p65 after LPS stimulation was detected in BV2 cells, peaking at 30 min post-exposure. However, pretreatment with (+)-JQ1 significantly reduced the entry of p65 into the nucleus. Taken together, these results indicate that (+)-JQ1 exerted a negative regulatory effect on LPS-induced NFκB signaling in microglia via the sequential modulation of the key events involved in the transcription of the proinflammatory genes. We then evaluated the phosphorylation of 2 up-stream NFκB related kinases, IKKα/β and IκBα. We found that LPS could significantly enhance the phosphorylation of IKKα/β and IκBα, followed by NFκB p65 phosphorylation itself. And (+)-JQ1 could obviously attenuate LPS induced signaling activation, from very early time point (30 min) (Fig. 3c). To further validate regulative effect of (+)-JQ1 in NFκB signaling, we than treated BV2 cells with p65 inhibitor PDTC after LPS stimulation, together with or without (+)-JQ1. Results showed that inhibition of p65 had the similar effect as JQ1 treatment, implying that JQ1 played the anti-inflammatory effect dependent on NFκB signaling (Fig. 3d). Thus, we may conclude that (+)-JQ1 attenuates LPS induced microglial activation and inflammation via IKKα/β-IκBα-NFκB signaling.

### MAPK signaling is targeted by (+)-JQ1

**Fig. 4:**
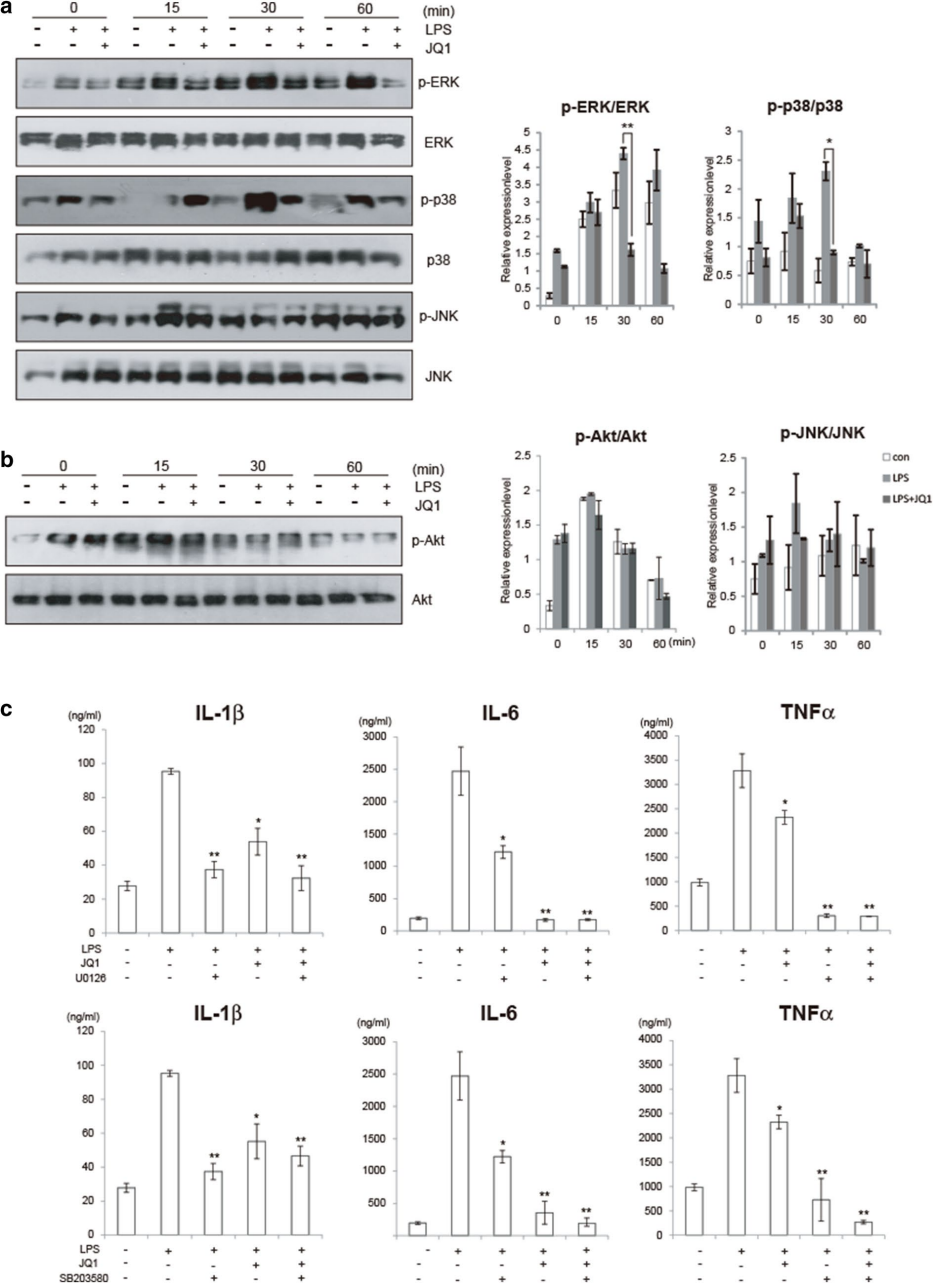
MAPK but not PI3 K signaling is targeted by (+)-JQ1. BV2 cells were pretreated with either (+)-JQ1 (400 nM) or DMSO for 30 min and subjected to LPS stimulation. The phosphorylation of ERK, p38, JNK (**a**) and Akt (**b**) as well as the total protein content, was analyzed by immunoblot. The relative intensity of a given band was normalized. Representative results from three independent experiments are depicted here. The relative intensity of a given band was normalized (n = 6). **c** BV2 cells were treated with LPS (100 ng/ml) or JQ1 (400 nM), together with or without U0126 (ERK inhibitor) or SB203580 (p38 inhibitor). 24 h later, cell culture medium were collected and IL-1β, IL-6 and TNFα were tested by ELISA (n = 3). The data are presented as the mean ± SEM (* *p *≤ 0.05, ** *p *≤ 0.01)

The three components of the MAPK (mitogen-activated protein kinase) family (ERK1/2, p38 and JNK) have been implicated in the transcription and production of proinflammatory factors and other effector molecules [46, 47]. We assessed the effects of (+)-JQ1 on LPS-induced MAPK activation in BV2 cells. As shown in Fig. 4a, treatment of microglia with LPS induced a marked increase in the phosphorylation of p38, ERK and JNK. However, (+)-JQ1 treatment significantly reduced those phosphorylations, especially to p38 and ERK. The phosphorylation of JNK was not related to JQ1 treatment according to our results. PI3K (phosphoinositide 3-kinase)/Akt signaling is another critically involved mediator in various biological activities, including the inflammatory response, cell growth, movement and survival [48, 49]. But, from our results, Akt phosphorylation did not seem to participate in the anti-inflammatory effect of (+)-JQ1 (Fig. 4b). Then BV2 cells were treated with ERK or p38 inhibitor U0126 or SB203580. Results showed that JQ1 treatment reduced LPS induced cytokine secretion, even stronger than treatment of ERK or p38 inhibitor (Fig. 4c), suggesting that JQ1 played the anti-inflammatory effect only partially dependent on MAPK signaling. MAPKs are critically involved in NFκB-mediated signaling and transcriptional activity. Thus, we speculate that (+)-JQ1 may mediate NFκB transcription at least partially via regulation of MAPK signaling.

### (+)-JQ1 attenuated LPS induced glial activation and neuroinflammation in vivo

**Fig. 5:**
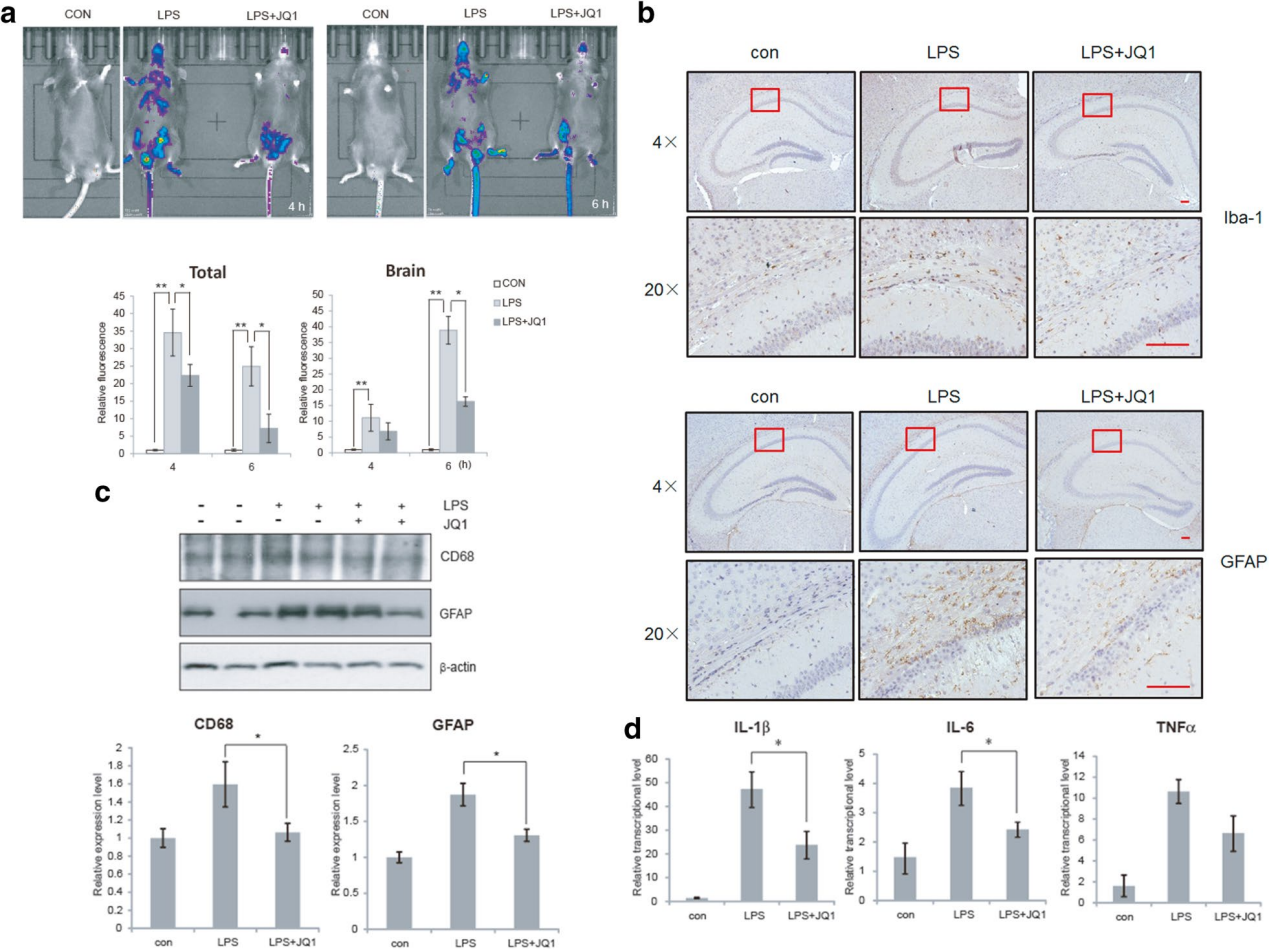
(+)-JQ1 reduces LPS induced neuroinflammation in vivo. **a** IL-1β-luc transgenic mice were intraperitoneally injected with LPS (2 μg in 2 μl), together with or without ICV injection of (+)-JQ1 (50 mg/kg). 4–6 h later, the in vivo fluorescence images were taken on and the fluorescence intensity was measured (n = 3). **b**, **c** Mice were ICV injected with LPS (2 μg in 2 μl), together with or without (+)-JQ1 (50 mg/kg). 4 h later, mice were sacrificed and brain tissue samples were collected. Reactive astrocytes (GFAP +) and microglia (Iba-1 +) were detected by IHC from brain tissue sections (scale bar: 100 μm) (**b**). GFAP (astrocyte) and CD68 (microglia) expression level were detected by immnoblot from brain tissue protein sample. immunoblot quantification was carried out by data normalization to β-actin level (**c**). Transcription levels of IL-1β, IL-6 and TNFα in the brain tissue were quantitatively analyzed by qRT-PCR. The data are presented as the mean ± SEM (n = 6, * *p *≤ 0.05)

On the basis of in vitro cell experiments, we have found that (+)-JQ1 had the anti-inflammatory effect. Thus, we perform further research by some in vivo approaches. GFP-IL-1β transgenic mice were applied and inflammation was induced by LPS IP injection. Fluorescence imaging results showed that (+)-JQ1 could significantly reduce LPS-induced inflammation in vivo (Fig. 5a), especially in the brain. Although we analyzed the fluorescence signal specifically for brain area, we cannot distinguish the signals between peripheral and central neuronal inflammation. To make it more focused on brain and neuroinflammation, ICV injection was performed and brain tissues were collected for neuroinflammation evaluation. We found that LPS injection could augment the activation of both astrocytes and microglia from IHC results (Fig. 5b). The expression levels of CD68 (microglia) and GFAP (astrocytes) increased (Fig. 5c) and transcription levels inflammatory cytokines IL-1β, IL-6 and TNFα were up-regluated (Fig. 5d). Then (+)-JQ1 treatment could dramatically reduce LPS induced glial activation and cytokine expression (Fig. 5b–d). All the evidence demonstrated that (+)-JQ1 attenuated LPS induced glial activation and neuroinflammation in vivo.

### (+)-JQ1 attenuated neuroinflammation via MAPK/NFkB signaling in vivo

**Fig. 6:**
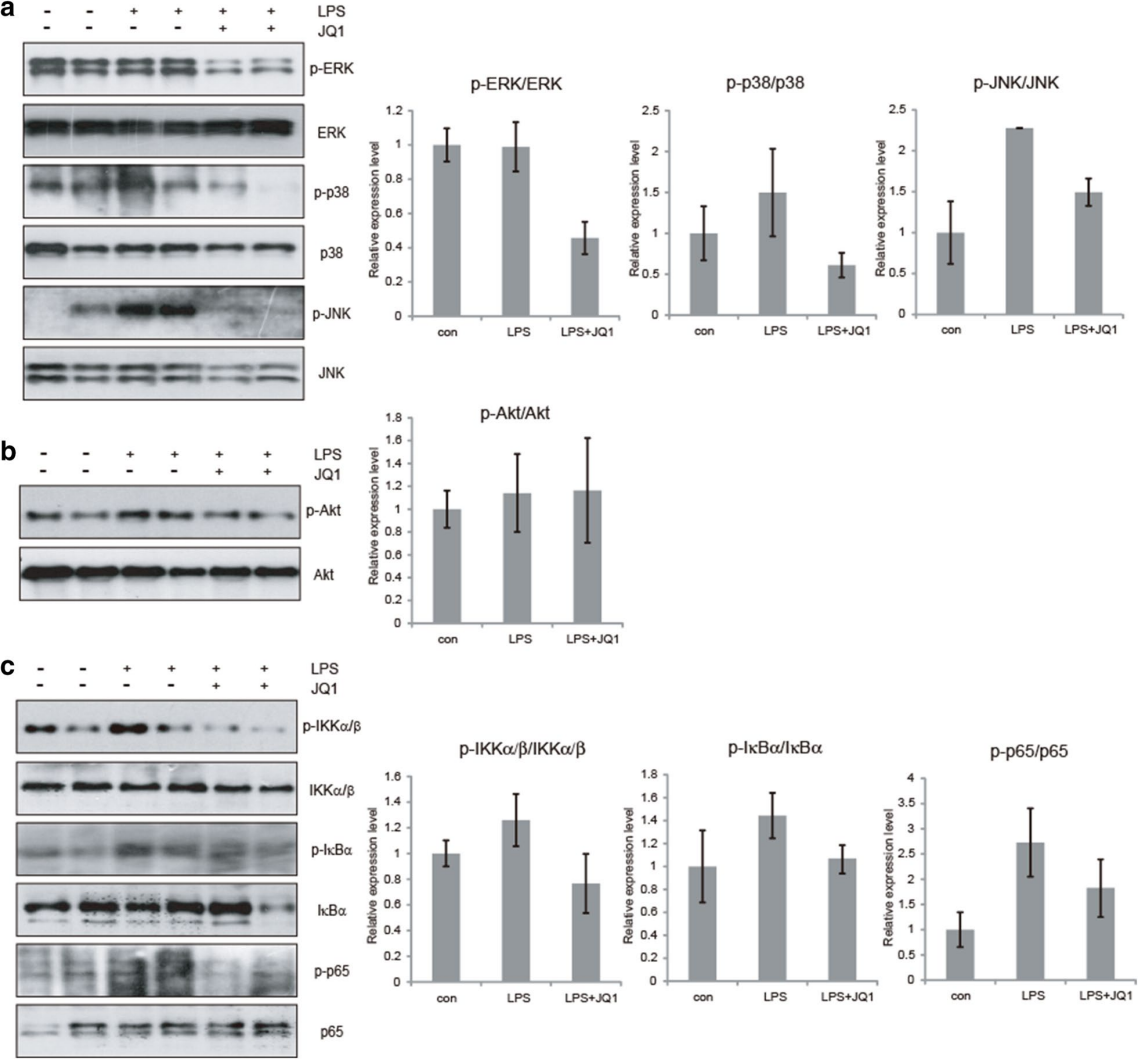
Anti-neuroinflmmation of (+)-JQ1 depends on MAPK/NFκB signaling in vivo. Mice were IVC injected with LPS (2 μg in 2 μl), together with or without (+)-JQ1 (50 mg/kg). 4 h later, mice were sacrificed and brain tissue samples were collected. The phosphorylation of p38, ERK1/2, JNK (**a**) and Akt (**b**) as well as the total protein content, was analyzed by immunoblot. Phosphorylation of IKKα/β (p-IKKα/β), IκBα (p-IκBα) and p65 (p-p65), as well as the total protein content, were analyzed by immnoblot (**c**). Representative results from three independent experiments are depicted here. The relative intensity of a given band was normalized. The data are presented as the mean ± SEM (n = 6)

**Fig. 7:**
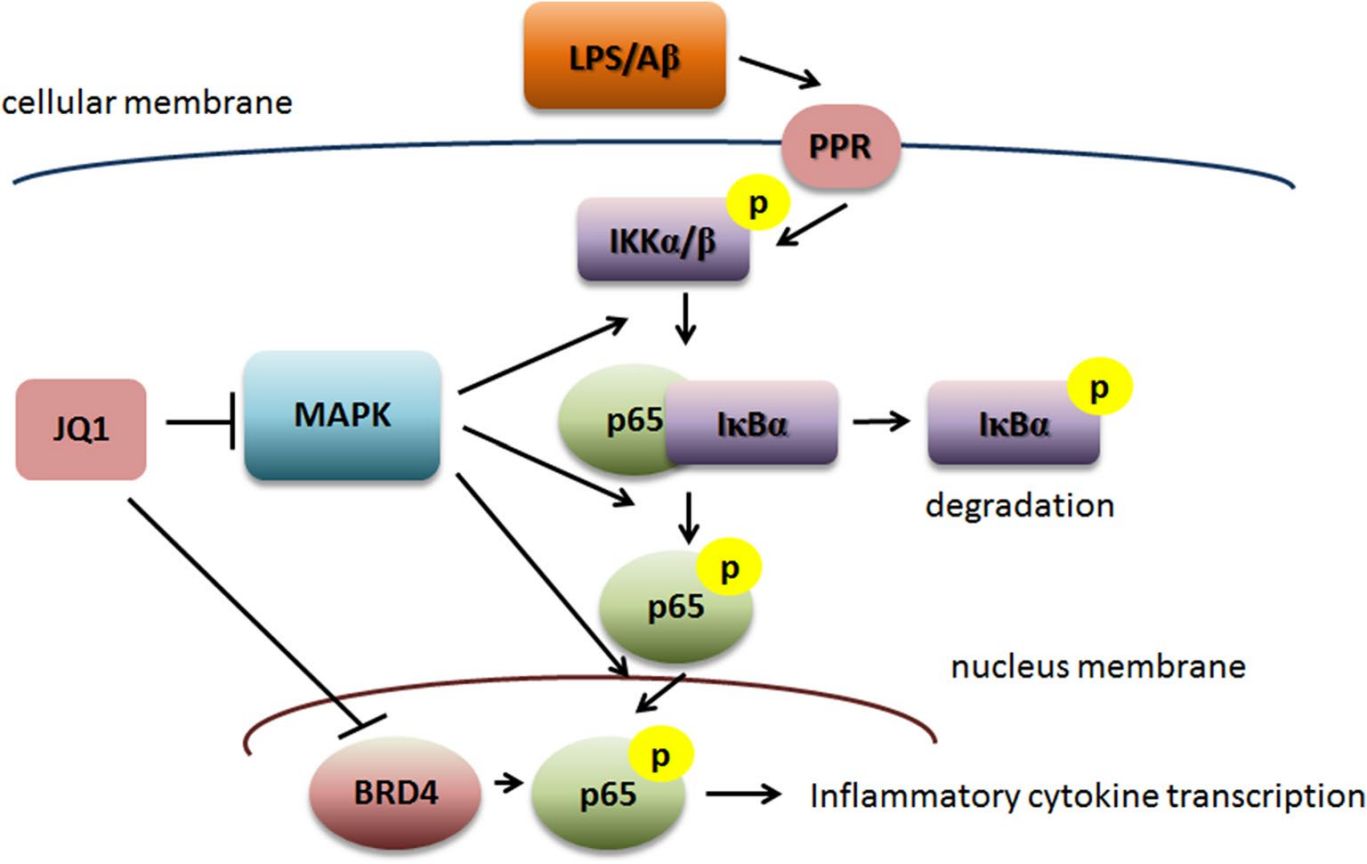
(+)-JQ1 attenuated LPS-induced microglial activation and transcription of proinflammatory cytokines via inhibition of NFκB translocation and MAPK signaling

Similar as studies in BV2 microglial cells, we also evaluate the activation of MAPK or PI3K/NFκB signaling from brain tissue. Immunoblot results showed that (+)-JQ1 could significantly attenuate the phosphorylation of MAPK signaling, including ERK, p38 and JNK (Fig. 6a). PI3K signaling and Akt phosphorylation seemed to play minor role in the effect of (+)-JQ1 (Fig. 6b). Then, the upregulted phosphorylation levels of NFκB related IKKα/β, IκBα and p65 induce by LPS injection could be also released by (+)-JQ1 treatment (Fig. 6c). All these data indicates that (+)-JQ1 reduces neuroinflmmation in vivo dependent on MAPK/NFκB signaling (Fig. 7).

## Discussion

Acute and chronic neuroinflammation have been implicated in contributing to the etiology of a variety of neurodegenerative diseases. Epidemiological and prospective population-based studies show an association between suppression of inflammation and reduced risk for AD [50]. Excessive and massive activation of microglia is thought to cause neuronal death and brain injury, most likely due to the production of high levels of cytotoxic and proinflammatory factors. The release of a variety of neuroinflammation related cytokines, like IL-1β and TNFα has been considered to contribute to the discrepancy of healthy neuronal function, further supporting the neuroinflammation hypothesis. Therefore, attenuation of microglial activation and neuroinflammation is considered to represent a potential strategy for the treatment of neurodegenerative diseases. In the current study, we found that (+)-JQ1 treatment could significantly reduce LPS induced microglial-activation, followed by attenuated transcription, expression and secretion of various inflammatory cytokines. It was proved that phosphorylation of MAPK signaling and nucleus relocalization of NFκB are crucial events in the anti-inflammatory process of (+)-JQ1 administration.

Although (+)-JQ1 was firstly developed as an anti-tumor agent, its anti-inflammatory effects were evaluated and studied in recent years. In vitro cell model experiments, (+)-JQ1 treatment has been verified to relieve many kinds of inflammation, like gastric, vascular endothelial and renal inflammation [20, 36, 37]. In animal experiments, (+)-JQ1 has also been proven to be effective in therapy many inflammatory diseases, like skin inflammation [34]. In CNS-related inflammation, it was reported that (+)-JQ1 treatment resulted in the downregulation of microglial inflammation related gene expression [40]. And in astrocytes (+)-JQ1 might dampen the proinflammatory activity induced by bryostatin-1 [51]. Similarly, in our current project, we found that acute (+)-JQ1 treatment in LPS activated BV2 cells could obviously inhibit the transcription and excretion of a series of inflammatory cytokines, including IL-1β, IL-6 and TNFα. In animal experiments, IP injection of (+)-JQ1 significantly reduced LPS induced IL-1β-GFP fluorescence, especially IL-1β triggered fluorescence in the brain (Fig. 5a). LPS acute treatment in mice resulted to activation of microglia (Iba-1 +) and astracytoes (GFAP +), which could be inhibited by (+)-JQ1 ICV injection (Fig. 5b, c). The upregulation of transcriptional levels of inflammatory cytokines induced by LPS was also attenuated by (+)-JQ1 treatment (Fig. 5d). Both in vitro and in vivo evidence revealed favorable anti-inflammatory effect of (+)-JQ1 against microglial activation.

The inflammatory response is a multi-step process characterized by the sequential activation of signaling molecules and transcription factors, which in turn trigger the expression of effector molecules. An array of cytokines, chemokines and other mediators are produced and released, some of which directly cause neuronal damage and dysfunction. NFκB signaling is one of most important and prevalent signaling pathways participating pattern recognition receptor (PPR) mediated cellular inflammatory response. Upon stimulation, NFκB (p65) translocates to the nucleus, where it acts as a master regulator of inflammatory genes. Activated NFκB interacts with and recruits coactivators, including BRD4, a reader of active gene-associated histone acetylation marks [52, 53]. BRD4 combination with NFκB (p65) may enhance the transcription of inflammatory genes. To elucidate the molecular mechanism underlying the anti-inflammatory function of (+)-JQ1 in microglia, we first evaluated its influence on NFκB activity. The results demonstrated that LPS induced NFκB activity was substantially suppressed 2 h after LPS treatment by (+)-JQ1 (Fig. 3a). The phosphorylation and relocalization of p65 were inhibited by (+)-JQ1 around 30 min after LPS treatment (Fig. 3b, c).This suppressive function of (+)-JQ1 appeared to be related to its modulation of the phosphorylation and degradation of IκBα 15–30 min after LPS treatment (Fig. 3c). Suppression of IκBα activation by (+)-JQ1 is likely caused by negative regulation of the upstream kinase IKK, as revealed by our study. Moreover, phosphorylation and nuclear translocation of p65 were also affected by (+)-JQ1. The similar results were also obtained from LPS-injected mouse. (+)-JQ1 treatment attenuated the phosphorylation of IKK/IκB and p65 in brain tissue sample. All these data are consistent with earlier work that shown BET inhibitors potently suppress the inflammatory transcriptional response, which might be related to a direct interaction between BRD4 and the acetylated p65 subunit of NFκB [18]. Thus, we hypothesize a model of sequential effects of (+)-JQ1 on NFκB activity in microglia.

The propagation of inflammatory mediators proved to be controlled by a complex network of signaling pathways. In addition to NFκB signaling, MAPK and PI3K were demonstrated to play an important role in the LPS-induced expression of proinflammatory cytokines. In earlier research concerning to (+)-JQ1 and anti-tumor effect, (+)-JQ1 was proved to play its role via PI3K/Akt signaling. In xenografts models of PTEN (phosphatase and tensin homolog)-positive endometrial cancer, (+)-JQ1 significantly upregulated the expression of PTEN, blocked the PI3K/Akt signaling pathway and suppressed tumor growth [54]. However, from our results, we found that, (+)-JQ1 may participate upstream of NFκB signaling via MAPK but not PI3 K signaling. The MAPK pathways consist of a family of highly conserved serine/threonine protein kinases that are believed to act as the regulators of key cellular processes including gene induction, cellular stress and inflammatory responses. There are three major classes of MAPKs in mammals, ERK, p38 and JNK [55]. We observed a reduction by (+)-JQ1 treatment in the phosphorylation ERK and p38 in LPS treated BV2 cells (Gig. 4A). Those results implied that the anti-inflammatory effect of (+)-JQ1 in micorglia and CNS may be partially dependent on MAPK but not PI3K signaling, specifically distinguished with tumor and peripheral inflammation. The inhibition of CNS inflammation remains some special characteristics. But the reduction of LPS induced cytokine secretion by (+)-JQ1 was stronger than treatment of ERK or p38 inhibitor (Fig. 4c), suggesting that JQ1 played the anti-inflammatory effect only partially dependent on MAPK signaling. (+)-JQ1 is traditionally a BRD4 inhibitor. Brd4 has been commonly recognized as transcription enhancer. Thus, it would be reasonable that MAPK/NFκB signaling inhibition by (+)-JQ1 could be an effective supplement to BRD4 directly inhibition.

Inhibition of NFκB mediated glial activation in animal PD models displayed an attenuation of neuronal damage and rescued memory impairments [56]. There is increasing interest in the search for novel compounds that target the critical glial inflammatory cytokine pathways and that display the potential to slow and even prevent neurodegenerative diseases [57–59]. Actually in animal experiments, (+)-JQ1 has been proven to reduce splenomegaly and neuroinflammation in the brain, together with a reduction of tau phosphorylation in the hippocampus and frontal cortex [42, 60]. However, evidence has been reported either support or oppose to the benefit effect of (+)-JQ1 on learning and memory function in AD mice [42, 60]. The specific explanation remains to be revealed. In our research, (+)-JQ1 could significantly reduce LPS induced neuroinflammation both in vitro and in vivo. All the data were achieved by acute treatment and cognitive function of mice were not evaluated. We speculate the possible reason for that (+)-JQ1 treatment did not improve cognitive function in AD mice could be the participation of BRD4 in neuron. Korb et al. [41] claimed that the loss of BRD4 function affects critical synaptic proteins, which results in memory deficits in mice but also decreases seizure susceptibility. To further research on (+)-JQ1 on neurodegenerative disease, both neuron and glia have to be considered in parallel. An optional strategy is to develop a selective BRD4 inhibitor, which could discriminate between neuron and glia. In that way, the inhibition of BRD4 in glia could be neuroprotective, while the enhancement of BRD4 may be benefit for brain function.

## Conclusions

In the present study, we provide evidence that demonstrates the immunomodulatory property of (+)-JQ1 in microglial activation and activity both in vitro and in vivo. We found that (+)-JQ1 could attenuate the LPS-induced production of proinflammatory cytokines and microglial activation in a dose- and time-dependent manner. The anti-inflammatory effect of (+)-JQ1 is closely associated with its modulation of NFκB activity and partially via MAPK signaling, which renders (+)-JQ1 as a promising therapeutic agent for microglia-mediated inflammatory disorders of the CNS (Fig. 7).

## Materials and methods

### Cell Culture and treatment

Mouse microglia BV2 cells were grown in high-glucose Dulbecco’s Modified Eagle’s Medium (DMEM) (Biological Industries, Iseral) supplemented with 10% fetal bovine serum (FBS) (Lanzhou Bailing Biotechnology, China) and penicillin/streptomycin (BBI Life Sciences, China). The cells were maintained in a humidified incubator (Sanyo, Japan) with a 95% air/5% CO_2_ atmosphere at 37 ^°^C. (+)-JQ1 (Nanjing Tianzhun, China) was dissolved in DMSO to a final concentration ranging from 0 to 10 μM. In the experiments indicated, the cells were pretreated with various concentrations of either (+)-JQ1 or DMSO 30 min prior to LPS (100 ng/ml) (Sigma-Alorich, USA)/Aβ (10 μM) (Sigma-Alorich, USA) treatment. Minomycine (2 μM) (Shyuanye, China) was administrated as the positive control. For NFκB signaling test, PDTC (p65 inhibitor), U0126 (ERK inhibitor) or SB203580 (p38 inhibitor) (Calbiochem, Germany) were treated with LPS or (+)-JQ1 for 24 h, followed by medium collection.

### Analysis of cell viability

Cell viability was measured via the MTT (3-(4,5-dimethylthiazol-2-yl)-2,5-diphenyltetrazolium bromide) assay (BBI Life Sciences, China). Briefly, BV2 cells were seeded in a 96-well plate at a density of 1 × 10^4^ cells per well in 200 μl of medium containing various concentrations of (+)-JQ1 (0–10 μM). After incubation for 24 h, the culture medium was removed, and MTT (0.5 mg/ml) was added to each well. After additional 4-h incubation, DMSO was added to dissolve the formazan crystals, and the absorbance was measured at 540 nm (Bio-Rad, USA). Wells without cells were used as blanks and were subtracted as the background from each sample. The results are expressed as the percentage of the control cells.

### In vivo mouse Immunofluorescence imaging

A transgenic mouse model bearing a reporter system luciferase under transcriptional control of a murine IL-1β promoter (IL-1β-luc) was applied to visualize the IL-1β response in the inflammation models which was induced by the LPS (1 μg/μl). 12 mice (male, 8 weeks) were randomly divided into 3 groups: control, LPS treatment and (+)-JQ1 treatment. 50 mg/kg of body weight of (+)-JQ1 was injected intraperitoneally 1 h before the LPS intracerebroventricular (ICV) injection. The in vivo images were taken by the OPT plus (shimadzu Co., Japan) 4–6 h after the LPS injected. The IL-1β-luc transgenic mice were obtained from Key Laboratory of Experimental Animal and Safety Evaluation, Zhejiang Academy of Medical Sciences.

### Intracerebroventricular (ICV) injection and mouse brain tissue preparation

Mice were anesthetized with pentobarbital sodium (80 mg/kg, i.p.) and placed on a stereotaxic frame (Stoelting). A stainless-steel micro-injector was stereotaxically implanted into the right lateral ventricle at 0.25 mm posterior to bregma, 1.0 mm lateral from midline and 3.0 mm vertically from the skull surface. LPS was dissolved in 0.01 M PBS (pH 7.4). LPS (2 μg in 2 μl) or sterile PBS (2 μl) was injected over 10 min. The micro-injector was left in place for 10 min to minimize back-flow, and then removed. The (+)-JQ1 (50 mg/kg of body weight) was injected intraperitoneally 1 h before LPS ICV injection. 4 h after the injection, mice were deeply anesthetized and perfused transcardially with 200 ml of 0.9% saline until blood was completely drained. Brains were extracted and hemisphere was fixed in 4% paraformaldehyde for 24 h at room temperature followed by series of dehydration and fixation for immunohistochemistry (IHC) detection. The other hemisphere was frozen immediately in liquid nitrogen and restored in − 80 °C freezer for later manipulation. Hemispheretissue was homogenized with TBS, sieved with 100 μm cell strainer (Falcon, USA) and centrifuged at 12,000*g* for 20 min at 4 °C. Supernatant was discarded and the pellet was further manipulated for mRNA and protein extraction.

### RNA isolation and RT-PCR (reverse transcription polymerase chain reaction)

Total RNA was isolated using RNAiso plus reagent (Sangon Biotech, China) according to the manufacturer’s protocol. Total RNA levels were then converted into cDNA using the High Capacity cDNA RT Kit (Takara, Japan).The quantification of gene expression was determined by realtime Q-PCR followed by SYBR Green PCR Master Mix (Cwbiotech, China) standard protocol. The relative expression levels were determined via the ΔΔCt method, using β-actin as an endogenous control. The primers used in the assays were listed in Table 1.

**Table 1:** Primers used in Q-PCR test

Genes	Primers
TNFα	Forward 5′-AAGGCCGGGGTGTCCTGGAG-3′
	Reverse 5′-AGGCCAGG TGGGGACAGCTC-3′
IL-1β	Forward 5′-AACCTCACCTACAGGGCGGACTTCA-3′
	Reverse 5′-TGTAATGAAAGACGGCACACC-3′
IL-6	Forward 5′-ATCCAGTTGCCTTCTTGGGACTGA-3′
	Reverse 5′-TAAGCCTCCGACTTGTGAAGTGGT-3′
β-actin	Forward 5′-TGTGATGGTGGGAATGGGTCAGAA-3′
	Reverse 5′-TGTGGTGCCAGATCTTCTCCATGT-3′

### Determination of cytokine expression

Concentrations of IL-1β, IL-6 and TNF-α were measured via ELISA kit (eBioscience, USA) according to the manufacture’s protocol. Briefly, after incubation, the cell culture medium was removed. Samples and standards were added to each well of microplate, which was precoated with primary antibody overnight. Each well was washed and incubated with the specific enzyme-linked polyclonal antibody for 2 h. The wells were washed to remove unbound antibody-enzyme reagent, and substrate solution was added to each well. After incubation for 20 min at room temperature, the enzyme reaction was stopped. Cytokine concentrations were determined by comparison of the optical density results with the standard curve.

### Immunoblot analysis

Cell lysates were prepared by lysing 5 × 10^6^ cells or 1 g brain tissue homogenate in lysis buffer RIPA (Solarbio, China) containing dissolved protease inhibitor (Beyotime, China) and phosphatase inhibitor (Sangon Biotech, China). Equal amounts of protein were separated using 10% SDS–polyacrylamide minigels and transferred to Immobilon PVDF membranes (Bio-Rad, USA). After blocking in Tris-buffered saline containing Tween-20 (TBST) and 5% bovine serum albumin (BSA), the membranes were incubated in the appropriate primary antibody followed by a secondary antibody conjugated to horseradish peroxidase (HRP) (MutiSciences, China). The signals were visualized using an ECL Western Blotting Kit (Millipore, USA). Blot density quantification was performed by software Quantity One (Bio-Rad, USA) and normalized to β-actin. Primary antibodies used in the current study were as follows: p-ERK, ERK, p-p38, p38, p-JNK, JNK, p-Akt, Akt, p-IKKα/β, IKKα/β, p-IκBα, IκBα, p-p65, p65 and β-actin were purchased from cell Signaling Technology, USA; CD68 was purchased from Abcam, UK; GFAP was purchase from Sigma-Alorich, USA.

### Assay of transcriptional activity of NFκB

To test NFκB-driven inflammatory cytokine transcriptional activity, BV2 cells were transfected with pκB-Luc and pRL-TK using lipofectamine 2000 transfection reagents (Invitrogen, USA). At 24 h after transfection, the cells were treated with (+)-JQ1 (400 nM), followed by stimulation using LPS. After incubation for 4 h, the cells were collected, and the luciferase activity was measured using dual luciferase assays (Promega, USA).

### Immunofluorescence staining and confocal microscopy

BV2 cells were seeded on slides at 30% confluence and were pretreated with either (+)-JQ1 (400 nM) or DMSO for 30 min. LPS was then added and maintained for the indicated period, after which the cells were collected, fixed with 100% methanol and permeabilized with 0.2% saponin. After blocking with 5% bovine serum, the cells were immunostained with a primary rabbit anti-p65 antibody overnight at 4 ^°^C, followed by treatment with a goat anti-rabbit IgG antibody conjugated to Texas Red (Life technologies, USA). Finally, after the nuclei were stained with DAPI (Life technologies, USA), the cells were mounted using Vectashield and analyzed via fluorescence confocal microscopy (Leica, Germany).

### Immohistochemistry (IHC)

Deparaffinized sections (8 μm) of brain were subjected to heat-induced antigen retrieval by incubation in 0.1 M EDTA, followed by washing with PBS. The slides were treated with 0.3% H_2_O_2_ and incubated with primary antibody overnight at 4 °C, followed by appropriate HRP-conjugated secondary antibody. The staining was developed using Fast 3,3′-diaminobenzidine tablet sets, while the sections were counterstained with hematoxylin and examined by light microscopy. The primary antibodies used in IHC were mouse anti-GFAP (Sigma) and rabbit anti-Iba-1 (Wako).

### Statistics

Unless otherwise indicated, all of the data are presented as the mean ± SEM. The statistical significance of the differences between two groups was analyzed using Student’s *t* test. A *p* value of 0.05 or less was considered to be statistically significant.

## Abbreviations

Aβ: β amyloid
AD: Alzheimer’s disease
BET: bromodomain and extrater-minal
BRD: bromodomain-containing protein
CNS: central nervous system
DMEM: Dulbecco’s Modified Eagle’s Medium
eRNA: enhancer RNA
FBS: fetal bovine serum
HRP: horseradish peroxidase
ICV: intracerebroventricular
IHC: immunohistochemistry
LPS: lipopolysaccharide
LTA: lipoteichoic acid
MAPK: mitogen-activated protein kinase
MS: multiple sclerosis
MTT: 3-(4,5-dimethylthiazol-2-yl)-2,5-diphenyltetrazolium bromide
NFκB: nuclear factor kappa-light-chain-enhancer of activated B cells
PD: Parkinson’s disease
PI3K: phosphoinositide 3-kinase
PPR: pattern recognition receptor
PTEN: phosphatase and tensin homolog
RA: rheumatoid arthritis
RT-PCR: reverse transcription polymerase chain reaction
TEFb: transcription elongation factor b
